# Prognostic Implications of Combined p53 and Mismatch-Repair Immunophenotypes in Uterine Carcinosarcoma

**DOI:** 10.3390/medicina62061135

**Published:** 2026-06-10

**Authors:** Emine Kilic Bagir, Umran Kucukgoz Gulec, Ilker Unal, Evin Kussever, Asli Sena Alagoz, Mehmet Ali Vardar, Derya Gumurdulu

**Affiliations:** 1 Department of Pathology, Faculty of Medicine, Cukurova University, 01330 Adana, Türkiye; evin_90line@hotmail.com (E.K.); gumurdulu@yahoo.com (D.G.); 2 Department of Obstetrics and Gynecology, Faculty of Medicine, Cukurova University, 01330 Adana, Türkiye; ukucukgoz@yahoo.com (U.K.G.); asliclkrrr@gmail.com (A.S.A.); mavardar@gmail.com (M.A.V.); 3 Department of Biostatistics, Faculty of Medicine, Cukurova University, 01330 Adana, Türkiye; ilkerun@cu.edu.tr

**Keywords:** uterine carcinosarcoma, mismatch repair deficiency, p53 expression, molecular classification, prognostic biomarkers, immunohistochemistry, tumor heterogeneity, immunotherapy

## Abstract

*Background and Objectives*: Uterine carcinosarcoma (UCS) is a rare and highly aggressive gynecologic malignancy with poor clinical outcomes and limited therapeutic options. This study investigated the prognostic significance of molecular subgroups defined by p53 expression and mismatch repair (MMR) status in UCS. *Materials and Methods*: This retrospective study included 51 patients with uterine carcinosarcoma who underwent surgical treatment between 2010 and 2023. Immunohistochemical analyses were performed to evaluate p53 expression (wild-type vs. aberrant) and MMR status (intact vs. deficient). Patients were classified into four molecular subgroups: p53wt/MMR-intact (*n* = 15), p53abn/MMR-intact (*n* = 24), p53wt/MMR-deficient (*n* = 9), and p53abn/MMR-deficient (*n* = 3). Clinicopathological characteristics, overall survival (OS), and disease-free survival (DFS) were analyzed. Additional component-specific analyses were performed for carcinomatous and sarcomatous tumor elements. *Results*: The median follow-up period was 34 months, and the overall mortality rate was 51.0%. Patients with p53wt/MMR-deficient tumors demonstrated the most favorable outcomes, with a mean OS of 92.9 ± 22.1 months and a mortality rate of 33.3%. In contrast, the p53abn/MMR-intact subgroup showed the poorest survival outcomes (mean OS: 54.4 ± 11.6 months; mortality rate: 62.5%). Although Kaplan–Meier survival analysis did not demonstrate statistically significant differences between molecular subgroups (*p* = 0.783), distinct prognostic trends were observed. Multivariate Cox regression analysis identified age and lymph node involvement as independent predictors of both OS and DFS. Component-specific analyses demonstrated significant associations between aberrant p53 expression in the carcinomatous component and epithelial subtype distribution (*p* = 0.025) as well as myometrial invasion patterns (*p* = 0.040). *Conclusions*: Combined p53/MMR-based immunohistochemical classification demonstrated distinct prognostic trends in uterine carcinosarcoma. These findings suggest that molecular stratification could support risk assessment and therapeutic decision-making in UCS. Larger prospective multicenter studies are warranted to validate these findings and clarify their potential clinical implications.

## 1. Introduction

Uterine carcinosarcoma (UCS), also known as malignant mixed Müllerian tumor (MMMT), is a rare and highly aggressive gynecologic malignancy, accounting for less than 5% of all uterine cancers [1]. Despite its rarity, UCS is responsible for a disproportionately high percentage of uterine-cancer-related deaths, with reported 5-year survival rates ranging from 30% to 40% [1,2]. Histologically, UCS is characterized by a biphasic architecture consisting of malignant epithelial (carcinomatous) and mesenchymal (sarcomatous) components, which has historically posed challenges in classification and therapeutic management [3].

Although the histological heterogeneity of UCS has long been recognized, recent advances in molecular pathology have provided new insights into its biological behavior. Increasing evidence suggests that UCS represents a form of metaplastic carcinoma rather than a true collision tumor [3,4]. Molecular studies have demonstrated that the carcinomatous and sarcomatous components typically share a common clonal origin and genetic alterations, indicating that the sarcomatous component may arise through epithelial–mesenchymal transition (EMT) of the epithelial component [3,4,5].

The 2020 World Health Organization (WHO) Classification of Female Genital Tumors [6] and large-scale genomic analyses such as The Cancer Genome Atlas (TCGA) [4] have significantly advanced our understanding of the molecular landscape of UCS. These studies have identified frequent alterations in key tumor suppressor genes, particularly TP53, which is mutated in approximately 90% of UCS cases [4,7,8,9,10]. In addition, defects in the DNA mismatch repair (MMR) system have been identified in a subset of UCS cases, which lead to microsatellite instability (MSI) and altered responsiveness to immune checkpoint inhibitors [11,12].

Despite these molecular advances, the prognostic implications of specific molecular alterations in UCS remain incompletely understood [9,10]. Most existing studies have focused on individual molecular markers in isolation, and only a limited number have evaluated the combined prognostic value of p53 status and MMR deficiency within a comprehensive molecular classification framework. Furthermore, the potential impact of molecular alterations within different tumor components (carcinomatous versus sarcomatous) has not been extensively investigated.

Current treatment strategies for UCS generally involve comprehensive surgical staging followed by adjuvant chemotherapy [13], with or without radiation therapy. Nevertheless, clinical outcomes remain poor, highlighting the need for improved prognostic stratification tools to guide therapeutic decision-making and identify patients who may benefit from targeted or immunotherapeutic approaches.

In this study, we aimed to (1) evaluate the prognostic significance of a molecular classification system combining p53 status and MMR deficiency in UCS; (2) analyze the association between molecular subgroups and clinicopathological characteristics; (3) investigate component-specific molecular alterations in the carcinomatous and sarcomatous elements; (4) identify independent prognostic factors for overall survival (OS) and disease-free survival (DFS); and (5) explore the potential clinical implications of molecular profiling for personalized treatment strategies in patients with UCS.

## 2. Materials and Methods

### 2.1. Study Population

This retrospective study included 51 patients with histologically confirmed uterine carcinosarcoma who underwent primary surgical treatment at our institution between January 2010 and December 2023. All patients underwent total abdominal hysterectomy with bilateral salpingo-oophorectomy, with or without lymph node dissection, based on intraoperative findings and clinical judgment. Patients who had incomplete medical records, insufficient tissue for immunohistochemical evaluation, or who had received neoadjuvant therapy were excluded.

This study was approved by the institutional ethics committee (Approval No. 134/2023) and conducted in accordance with the Declaration of Helsinki. Due to the retrospective nature of this study, the requirement for informed consent was waived.

### 2.2. Histopathological Evaluation

All surgical specimens were reviewed by experienced gynecologic pathologists. Histological diagnoses were established according to the World Health Organization (WHO) Classification of Female Genital Tumors. Tumors were staged according to the International Federation of Gynecology and Obstetrics (FIGO) 2009 staging system.

Histopathological assessment included evaluation of the epithelial and sarcomatous tumor components, epithelial histological subtype (endometrioid grade 3, serous, or other), sarcomatous subtype, depth of myometrial invasion, lymphovascular invasion (LVI), lymph node involvement (LNI), and FIGO stage. Tumors were categorized according to the depth of myometrial invasion (<50% or ≥50%) and the presence of a polypoid growth pattern.

For component-specific analyses, tumors were additionally categorized according to the dominant histological component (carcinomatous-dominant or sarcomatous-dominant) based on the relative proportion of each component observed during histopathological examination. The dominant component was defined as the component comprising more than 50% of the tumor volume. Clinicopathological data were obtained from pathology reports and institutional medical records.

### 2.3. Immunohistochemical Analysis

Immunohistochemical evaluation was performed on 5 μm thick sections obtained from formalin-fixed, paraffin-embedded (FFPE) tissue blocks using an automated BenchMark XT staining platform (Ventana Medical Systems, Tucson, AZ, USA) according to the manufacturer’s instructions. Antigen detection was carried out using the iView Blue Detection Kit (Ventana, Oro Valley, AZ, USA).

The following primary antibodies were used: p53 (clone DO7, Cell Marque, Merck SA, München, Germany), MSH2 (clone G219-1129, Cell Marque, Merck SA, München, Germany), MSH6 (clone 44, Cell Marque, Merck SA, München, Germany), MLH1 (clone G168-728, Cell Marque, Merck SA, München, Germany), and PMS2 (clone IHK412, GenomeMe, Richmond, BC, Canada). Immunostaining procedures were performed using the ISH Protease 2 protocol from the BenchMark XT automated staining system.

Mismatch repair (MMR) protein expression was evaluated by assessing nuclear staining in tumor cells. Loss of expression of one or more MMR proteins (MLH1, PMS2, MSH2, or MSH6) in tumor cells, in the presence of intact internal positive controls, including stromal and inflammatory cells, was interpreted as a mismatch repair deficiency (MMRd). Tumors demonstrating retained nuclear staining for all four proteins in at least 1% of tumor cells were classified as MMR-intact.

p53 expression patterns were classified as wild-type (p53wt) or abnormal/aberrant (p53abn). Wild-type expression was defined as heterogeneous nuclear staining of variable intensity in tumor cells. Aberrant p53 expression was defined as either diffuse strong nuclear staining in >80% of tumor cells (overexpression pattern) or complete absence of nuclear staining (null pattern). Cases were subsequently classified into four molecular subgroups based on both p53 expression and MMR status.

All immunohistochemical slides were independently evaluated by two experienced pathologists blinded to the clinical outcomes. Any discrepancies were resolved by consensus review.

### 2.4. Molecular Subgroup Classification

Based on both p53 and mismatch repair (MMR) statuses, tumors were classified into four molecular subgroups: p53wt/MMR-intact, p53abn/MMR-intact, p53wt/MMR-deficient, and p53abn/MMR-deficient. This classification system enabled evaluation of the combined effects of p53 abnormalities and MMR deficiency on clinicopathological characteristics and clinical outcomes. Additional analyses were performed to evaluate p53 expression separately in the carcinomatous and sarcomatous components and to further assess the association between MMR status and clinicopathological parameters.

### 2.5. Clinical Data Collection

Clinical and pathological data were retrieved from electronic medical records and pathology reports. The collected variables included age at diagnosis, body mass index (BMI), parity, menopausal status, comorbidities, tumor stage, histological subtypes, LVI, LNI, depth of myometrial invasion, treatment modalities (surgery, chemotherapy, and radiotherapy), follow-up duration, disease recurrence, and survival status.

### 2.6. Statistical Analysis

Descriptive statistics were used to summarize patient characteristics. Continuous variables are expressed as the mean ± standard deviation (SD) and compared using Student’s t-test or the Mann–Whitney U test, as appropriate. Categorical variables are presented as frequencies and percentages and were compared using the chi-square test or Fisher’s exact test.

Overall survival (OS) was defined as the time from the date of initial surgery to death from any cause or to the last follow-up. Disease-free survival (DFS) was defined as the time from the date of initial surgery to the date of disease recurrence or the last follow-up.

Survival curves were estimated using the Kaplan–Meier method, and differences between groups were assessed using the log-rank test.

Univariate and multivariate Cox proportional hazards regression analyses were performed to identify independent prognostic factors for OS and DFS. Variables with *p* < 0.10 in the univariate analysis were included in the multivariate model. Hazard ratios (HRs) with 95% confidence intervals (CIs) were calculated.

All statistical tests were two-sided, and *p* < 0.05 was considered statistically significant. Statistical analyses were performed using SPSS version 25.0 (IBM Corp., Armonk, NY, USA).

Due to the limited sample size and number of events, the number of variables included in the multivariate Cox regression model was restricted to avoid overfitting.

## 3. Results

### 3.1. Patient Characteristics and Molecular Subgroup Distribution

A total of 51 patients with uterine carcinosarcoma were included in this study. The mean age at diagnosis was 63.2 ± 10.3 years (range: 39–85 years), and the median follow-up duration was 34 months (range: 3–156 months), during which 26 deaths (51.0%) were observed. Most patients were postmenopausal (94.1%), and 70.6% of patients had at least one comorbidity. The mean body mass index (BMI) was 29.2 ± 7.9 kg/m^2^.

The distribution of patients across the four predefined molecular subgroups was as follows: *n* = 15 (29.4%) in p53wt/MMR-intact, *n* = 24 (47.1%) in p53abn/MMR-intact, *n* = 9 (17.6%) in p53wt/MMR-deficient, and *n* = 3 (5.9%) in p53abn/MMR-deficient. The p53abn/MMR-intact subgroup represented the largest proportion of cases.

### 3.2. Clinicopathological Characteristics According to Molecular Subgroups

The clinicopathological characteristics according to molecular subgroup are summarized in Table 1. No significant differences were observed among the four groups with respect to age at diagnosis (*p* = 0.753), BMI (*p* = 0.373), parity, menopausal status, or comorbidity status. Lymphovascular invasion (LVI) was present in 73% of p53wt/MMR-intact tumors, 67% of p53abn/MMR-intact tumors, 100% of p53wt/MMR-deficient tumors, and 67% of p53abn/MMR-deficient tumors (*p* = 0.201). Lymph node involvement (LNI) was observed in these groups at rates of 27%, 25%, 44%, and 67%, respectively (*p* = 0.365).

Myometrial invasion depth and FIGO stage distributions were similar across molecular subgroups (*p* = 0.201 and *p* = 0.777, respectively). Most patients presented with FIGO stage IB or higher.

### 3.3. Immunohistochemical and Component-Specific Analyses

Separate component-based analyses were performed for p53 and MMR status in both the carcinomatous and sarcomatous elements. Analysis of p53 status within the carcinomatous component demonstrated significant associations with epithelial subtype distribution and myometrial invasion (Table S1). Tumors with aberrant p53 expression showed a higher prevalence of serous carcinoma (47.6%) compared with those with wild-type p53 expression (13.3%), whereas endometrioid grade 3 histology was more frequent in p53wt tumors (66.7% vs. 38.1%) (*p* = 0.025). Myometrial invasion patterns also differed significantly between the groups (*p* = 0.040), with ≥50% invasion observed more frequently in p53wt tumors.

**Figure 1:**
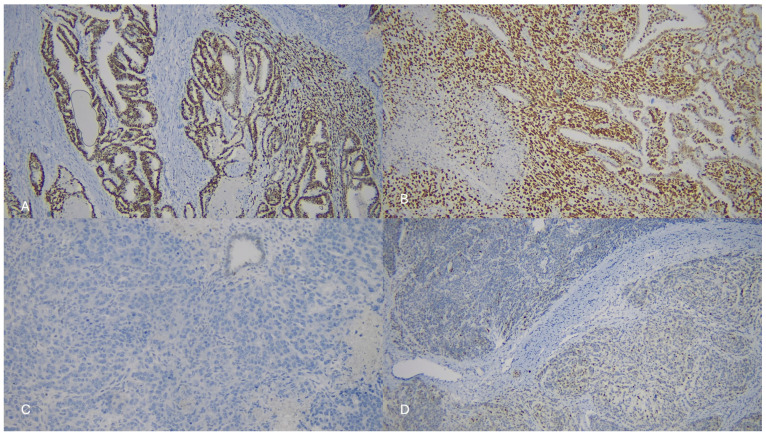
Immunohistochemical p53 staining patterns in uterine carcinosarcoma. (**A**) Aberrant p53 overexpression pattern in the epithelial (carcinomatous) component and sarcomatous component demonstrating diffuse strong nuclear staining (×200 magnification). (**B**) Aberrant p53 overexpression pattern in the sarcomatous component showing diffuse strong nuclear positivity (×100 magnification). (**C**) Aberrant p53 null-type staining pattern characterized by complete absence of nuclear staining in tumor cells (×200 magnification). (**D**) Wild-type p53 expression pattern demonstrating heterogeneous nuclear staining with variable intensity (×100 magnification).

In contrast, analysis of the sarcomatous component revealed no significant associations between p53 status and clinicopathological parameters (Table S2). The evaluation of overall p53 status across both tumor components showed similar trends, but they did not reach statistical significance (Table S3). Representative immunohistochemical p53 staining patterns are shown in Figure 1.

**Figure 2:**
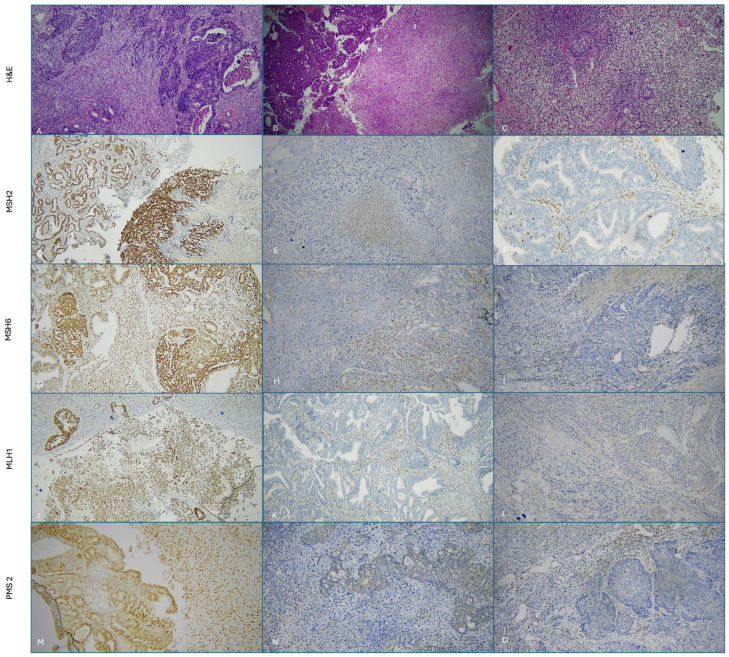
Representative hematoxylin–eosin (H&E) and mismatch repair (MMR) immunohistochemical staining patterns in uterine carcinosarcoma. (**A**–**C**) Hematoxylin–eosin (H&E)-stained sections demonstrating representative biphasic morphology of uterine carcinosarcoma. (**D**) Preserved nuclear MSH2 expression consistent with MMR-intact status. (**E**) Loss of MSH2 nuclear expression in the sarcomatous component consistent with mismatch repair deficiency (MMRd). (**F**) Loss of MSH2 nuclear expression in the carcinomatous component consistent with MMR deficiency. (**G**) Preserved nuclear MSH6 expression consistent with MMR-intact status. (**H**) Loss of MSH6 nuclear expression in the sarcomatous component. (**I**) Loss of MSH6 nuclear expression in the carcinomatous component. (**J**) Preserved nuclear MLH1 expression consistent with MMR-intact status. (**K**) Loss of MLH1 nuclear expression in the carcinomatous component. (**L**) Loss of MLH1 nuclear expression involving both carcinomatous and sarcomatous components. (**M**) Preserved nuclear PMS2 expression consistent with MMR-intact status. (**N**) Loss of PMS2 nuclear expression in the sarcomatous component. (**O**) Loss of PMS2 nuclear expression in the carcinomatous component. Magnifications: ×100 and ×200. Internal control cells retained positive nuclear staining.

No statistically significant association was observed between MMR status and clinicopathological parameters in the carcinomatous component (Table S4). The MMR groups did not show any significant differences in the other clinicopathological parameters. A trend toward improved survival was observed in MMR-deficient tumors, although this finding did not reach statistical significance (*p* = 0.067) (Table S5). When tumors were categorized simply as MMR-intact versus MMR-deficient, no significant differences were identified (Table S6). Representative hematoxylin–eosin (H&E) and mismatch repair (MMR) immunohistochemical staining patterns are presented in Figure 2.

### 3.4. Survival Outcomes and Prognostic Factors

**Figure 3:**
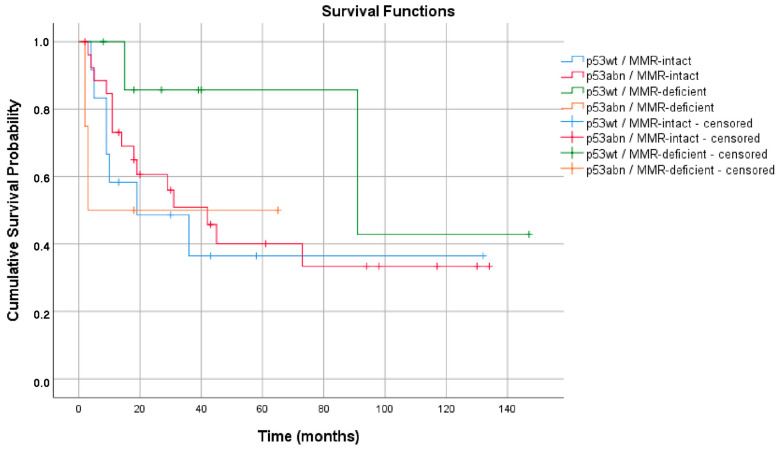
Kaplan–Meier overall survival (OS) curves of p53/MMR molecular subgroups.

Kaplan–Meier analysis demonstrated differences in overall survival among the molecular subgroups; however, these differences did not reach statistical significance (log-rank *p* = 0.783) (Table 2, Figure 3). Patients with p53wt/MMR-deficient tumors showed the most favorable outcomes, with a mean overall survival (OS) of 92.9 ± 22.1 months and a median OS of 91.0 months. The p53wt/MMR-intact subgroup showed intermediate survival outcomes (mean OS: 69.4 ± 16.5 months; median OS: 36.0 months). The p53abn/MMR-intact subgroup demonstrated the poorest outcomes, with a mean OS of 54.4 ± 11.6 months and a median OS of 31.0 months. The p53abn/MMR-deficient subgroup included three patients and had a mean OS of 44.0 ± 17.1 months.

**Figure 4:**
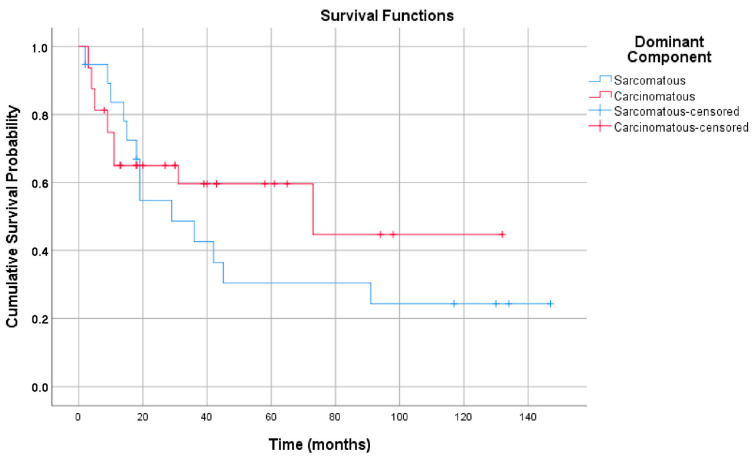
Kaplan–Meier overall survival curves according to dominant tumor component (carcinomatous-dominant vs. sarcomatous-dominant) in uterine carcinosarcoma.

Across the entire cohort, the mean OS was 69.2 ± 9.9 months, and the median OS was 42.0 months, with an overall mortality rate of 51.0%. Kaplan–Meier analysis stratified by dominant tumor component demonstrated survival differences between carcinomatous-dominant and sarcomatous-dominant tumors; however, these differences did not reach statistical significance (Figure 4).

In the univariate Cox regression analysis, age at diagnosis (HR = 1.059, 95% CI: 1.012–1.109, *p* = 0.014) and lymph node involvement (HR = 2.639, 95% CI: 1.170–5.952, *p* = 0.019) were significantly associated with OS. In the multivariate analysis (Table 3), both age at diagnosis (HR = 1.059, 95% CI: 1.012–1.109, *p* = 0.014) and lymph node involvement (HR = 2.639, 95% CI: 1.170–5.952, *p* = 0.019) remained independent predictors of survival.

**Figure 5:**
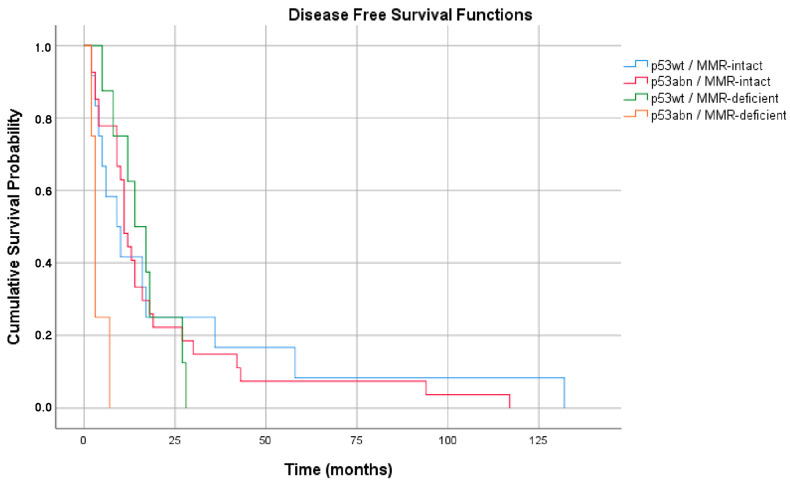
Kaplan–Meier disease-free survival (DFS) curves of p53/MMR molecular subgroups.

Kaplan–Meier analysis for disease-free survival (DFS) also demonstrated differences among the molecular subgroups (Figure 5). Similar results were observed for DFS in the multivariate analysis. In the multivariate analysis (Table 4), age at diagnosis (HR = 1.034, 95% CI: 1.003–1.065, *p* = 0.030) and lymph node involvement (HR = 2.630, 95% CI: 1.386–4.990, *p* = 0.003) were identified as independent predictors.

## 4. Discussion

This comprehensive immunohistochemical molecular profiling study of 51 patients with uterine carcinosarcoma suggests that combined assessment of p53 and mismatch repair (MMR) status may identify prognostically distinct subgroups among these patients. Our four-group molecular classification system (p53wt/MMR-intact, p53abn/MMR-intact, p53wt/MMR-deficient, and p53abn/MMR-deficient) identified distinct survival patterns, with mean overall survival times ranging from 44 to 92.9 months across subgroups. Furthermore, our component-specific analyses provide additional insights into the molecular complexity of uterine carcinosarcoma and its relationship with clinical outcomes. To our knowledge, this study is one of the few analyses to evaluate component-specific molecular alterations in uterine carcinosarcoma. Importantly, most previous studies have evaluated p53 or MMR alterations in isolation, whereas this study examined their combined prognostic impact within a unified molecular classification framework for uterine carcinosarcoma.

The distribution of molecular subgroups in our cohort reflects the known molecular landscape of UCS [4,14]. The p53abn/MMR-intact subgroup represented the largest proportion (47.1%), consistent with the high frequency of p53 mutations in UCS reported in TCGA studies [4,8,9,10]. This observation is also consistent with recent molecular classification studies demonstrating that uterine carcinosarcomas are predominantly represented by abnormal p53 molecular subtypes [15]. The relatively high proportion of MMR-deficient cases (p53wt/MMR-deficient and p53abn/MMR-deficient accounted for 21.5%) is notable and may reflect inclusion of both Lynch syndrome-associated and sporadic MMR-deficient cases.

Our survival analysis revealed that the p53wt/MMR-deficient subgroup exhibited the most favorable prognosis (mean OS of 92.9 months, 33.3% mortality), while the p53abn/MMR-intact subgroup showed the poorest prognosis (mean OS of 54.4 months, 62.5% mortality). This 38.5-month difference in mean survival suggests a potentially relevant prognostic trend. The intermediate survival observed in the p53wt/MMR-intact subgroup (mean OS of 69.4 months, 46.7% mortality) and the relatively favorable outcomes in the small p53abn/MMR-deficient subgroup (mean OS of 44 months, 33.3% mortality) suggest that MMR deficiency may attenuate the adverse prognostic impact associated with aberrant p53 expression, although this conclusion is based on a very limited number of cases and larger studies are needed to confirm this observation.

The favorable prognosis of MMR-deficient tumors, particularly those with wild-type p53 expression, likely reflects their enhanced immunogenicity [11,12,16] due to their high tumor mutational burden and neoantigen expression. This biological characteristic not only influences the natural disease course but also suggests potential responsiveness to immune checkpoint inhibitors, as demonstrated in other MMR-deficient malignancies [12,17,18]. Therefore, identification of MMR-deficient tumors in uterine carcinosarcoma may have direct therapeutic implications, particularly in the era of biomarker-driven immunotherapy. Recent molecular analyses have similarly reported distinct clinical outcomes among UCS molecular subgroups, supporting the prognostic relevance of molecular classification of these tumors [19].

Our component-specific analyses provide additional insights into the biological behavior of uterine carcinosarcoma. The significant association between aberrant p53 expression in the carcinomatous component and serous histology (*p* = 0.025; Supplementary Table S1) is particularly noteworthy. p53abn tumors showed a markedly higher prevalence of serous carcinoma compared with p53wt tumors (Supplementary Table S1). This observation is consistent with the well-established role of p53 mutations in uterine serous carcinoma [4,20] and suggests that p53 status in the carcinomatous component may influence epithelial differentiation toward more aggressive phenotypes.

Notably, p53wt tumors exhibited deeper myometrial invasion compared with p53abn tumors (*p* = 0.040). Although the biological explanation for this finding remains uncertain, it may reflect differences in tumor growth patterns. p53wt tumors may exhibit more infiltrative behavior, whereas p53abn tumors may grow in a more expansile or polypoidal fashion.

In contrast, the p53 status in the sarcomatous component did not show any significant associations with clinicopathological parameters. This finding suggests that molecular alterations within the carcinomatous component may have a greater influence on clinicopathological characteristics than those in the sarcomatous component. However, given the limited sample size and the biological complexity of uterine carcinosarcoma, further studies are required to clarify the relative contribution of each component to tumor behavior and prognosis.

Additional analyses of MMR status revealed a complex relationship with clinicopathological features. Although no statistically significant associations were identified between MMR status and most clinicopathological parameters, the trend toward improved survival observed in the sarcomatous component analysis of MMR-deficient tumors (*p* = 0.067) further supports the hypothesis that MMR deficiency may confer a prognostic benefit through enhanced tumor immunogenicity and increased responsiveness to antitumor immune surveillance.

The lack of significance in the simplified MMR analysis (intact versus deficient only) suggests that molecular risk stratification may require integrating multiple markers rather than relying on a single biomarker.

Our multivariate Cox regression analyses identified age at diagnosis and lymph node involvement as independent prognostic factors for both overall survival and disease-free survival. The consistency of these findings across both endpoints strengthens their clinical relevance. Each additional year of age increased the mortality risk by approximately 6% (HR = 1.059 for OS and HR = 1.034 for DFS), which may reflect differences in treatment tolerance, comorbidities, or tumor biology. Lymph node involvement conferred a 2.6-fold higher risk of death (HR = 2.639 for OS and 2.630 for DFS), which underscores the importance of comprehensive surgical staging and careful postoperative management in patients with nodal metastasis.

Although molecular classification did not reach statistical significance as an independent prognostic factor in the multivariate analysis, likely due to the relatively small cohort size, the observed survival differences between molecular subgroups may still be clinically informative. The observed difference in survival between the p53wt/MMR-deficient and p53abn/MMR-intact subgroups suggests that with larger cohorts, molecular classification may emerge as an independent prognostic indicator. These findings support the concept that molecular classification may complement traditional clinicopathological factors in refining risk stratification for patients with uterine carcinosarcoma.

Our findings are broadly consistent with previous studies investigating the molecular landscape of uterine carcinosarcoma [4,7,8,9,10]. TCGA studies [4] have highlighted the high prevalence of p53 mutations in these tumors and identified molecular subtypes with prognostic implications. However, most previous investigations have evaluated p53 and MMR status separately rather than in combination, and few studies have explored component-specific molecular alterations.

The favorable prognosis of MMR-deficient tumors observed in this study is also consistent with reports in other gynecologic malignancies [11,21,22], particularly endometrial carcinoma, where MMR deficiency has been associated with improved outcomes [20] and responsiveness to immunotherapy. Recent studies have demonstrated that MMR-deficient uterine carcinosarcomas exhibit distinct clinicopathological and molecular characteristics compared with conventional carcinosarcomas [23]. The approval of immune checkpoint inhibitors such as pembrolizumab for MMR-deficient solid tumors [17,18] further emphasizes the potential clinical importance of molecular classification in uterine carcinosarcoma.

From a therapeutic perspective, the molecular classification framework proposed in this study may have several important clinical implications. Patients with MMR-deficient tumors are potential candidates for immune checkpoint inhibitor therapy. Conversely, patients in the p53abn/MMR-intact subgroup, who demonstrated the poorest survival outcomes, may benefit from more aggressive adjuvant treatments or novel targeted therapies. The favorable prognosis observed in p53wt/MMR-deficient tumors also raises the possibility of treatment de-escalation for carefully selected patients, particularly those of advanced age or with significant comorbidities.

This study has several strengths, including the comprehensive molecular classification that combines p53 and MMR status, the component-specific evaluation of carcinomatous and sarcomatous elements, and the analysis of overall and disease-free survival. However, several limitations should be acknowledged. The relatively small number of patients in the p53abn/MMR-deficient subgroup may have limited the statistical power of the subgroup analyses and reduced the ability to detect significant survival differences. Furthermore, the molecular classification was based primarily on immunohistochemical assessment without comprehensive genomic sequencing, POLE mutation analysis [24,25,26], or microsatellite instability testing, thereby limiting complete TCGA/ProMisE molecular classification. Finally, the absence of detailed treatment stratification may have introduced confounding in the survival analyses.

Future studies should validate these findings in larger multicenter cohorts and incorporate comprehensive genomic profiling, including POLE sequencing, and validated molecular classification systems such as ProMisE [27,28] in combination with immunohistochemical classification. Prospective clinical trials evaluating immunotherapy in MMR-deficient uterine carcinosarcoma and targeted therapies for the molecular subgroups associated with poor prognosis could further refine personalized treatment strategies. A better understanding of the molecular evolution of the carcinomatous and sarcomatous components, particularly when integrated with updated staging systems such as FIGO 2023 [29], may provide additional insights into the biology and therapeutic vulnerabilities of uterine carcinosarcoma.

## 5. Conclusions

This study’s findings suggest that combined p53/MMR-based molecular stratification may provide clinically meaningful prognostic information in uterine carcinosarcoma. Patients with p53wt/MMR-deficient tumors had the most favorable survival, whereas patients with p53abn/MMR-intact tumors had the poorest survival. Component-specific analyses further suggest that p53 alterations in the carcinomatous component may play a central role in tumor behavior. Age at diagnosis and lymph node involvement were identified as independent prognostic factors for both overall and disease-free survival. These findings support the integration of molecular profiling into UCS risk stratification and highlight potential therapeutic implications, particularly the use of immune checkpoint inhibitors to treat MMR-deficient tumors. Larger prospective studies are required to validate these results and further improve personalized therapeutic approaches in uterine carcinosarcoma.

## Figures and Tables

**Table 1:** Comparison of demographic, clinical, and pathological outcomes of subgroups.

	p53wt/MMR-Intact	p53abn/MMR-Intact	p53wt/MMR-Deficient	p53abn/MMR-Deficient	*p*
Age at Diagnosis (Mean ± SD)	62.8 ± 13.8	64.3 ± 7.3	60.8 ± 10	66.7 ± 10.7	0.753
BMI (Mean ± SD)	28.6 ± 8.8	29.4 ± 7.2	27.1 ± 8.2	36.3 ± 8.1	0.373
Menopause Status, *n* (%)					0.646
Pre-/Perimenopausal	2 (13%)	1 (4%)	0 (0%)	0 (0%)	
Postmenopausal	13 (87%)	23 (96%)	9 (100%)	3 (100%)	
LVI, *n* (%)					0.201
Negative	4 (27%)	8 (33%)	0 (0%)	1 (33%)	
Positive	11 (73%)	16 (67%)	9 (100%)	2 (67%)	
LNI, *n* (%)					0.365
Negative	11 (73%)	18 (75%)	5 (56%)	1 (33%)	
Positive	4 (27%)	6 (25%)	4 (44%)	2 (67%)	
Myometrial Invasion, *n* (%)					0.201
<50% or Polyp	4 (27%)	8 (33%)	0 (0%)	1 (33%)	
≥50%	11 (73%)	16 (67%)	9 (100%)	2 (67%)	
Stage, *n* (%)					0.777
1A	3 (20%)	5 (21%)	0 (0%)	1 (33%)	
1B	5 (33%)	9 (38%)	3 (33%)	0 (0%)	
2	2 (13%)	2 (8%)	0 (0%)	0 (0%)	
3	4 (27%)	6 (25%)	5 (56%)	2 (67%)	
4	1 (7%)	2 (8%)	1 (11%)	0 (0%)	
Status, *n* (%)					0.455
Ex	7 (47%)	15 (63%)	3 (33%)	1 (33%)	
Alive	8 (53%)	9 (37%)	6 (67%)	2 (67%)	

LVI: lymphovascular invasion; LNI: lymph node involvement; BMI: body mass index; SD: standard deviation.

**Table 2:** Kaplan–Meier analysis for overall survival (OS).

	*n*	Events (%)	Censored (%)	Mean Survival (Months)	95% CI	Median Survival (Months)	*p*
p53wt/MMR-intact	15	7 (47)	8 (53)	69.4 ± 16.5	37.0–101.8	36.0	0.783
p53abn/MMR-intact	24	15 (63)	9 (38)	54.4 ± 11.6	31.6–77.2	31.0	
p53wt/MMR-deficient	9	3 (33)	6 (67)	92.9 ± 22.1	49.5–136.3	91.0	
p53abn/MMR-deficient	3	1 (33)	2 (67)	44.0 ± 17.1	10.4–77.6	NA	
Overall	51	26 (51)	25 (49)	69.2 ± 9.9	49.7–88.7	42.0	

NA, not applicable.

**Table 3:** Multivariate Cox regression analysis for overall survival (OS).

	*p*	Hazard Ratio	95.0% CI Lower–Upper
Age at diagnosis	**0.014**	1.059	1.012–1.109
LNI	**0.019**	2.639	1.170–5.952

LNI: lymph node involvement. Statistically significant data is highlighted in bold.

**Table 4:** Multivariate Cox regression analysis for disease-free survival (DFS).

	*p*	Hazard Ratio	95.0% CI Lower–Upper
Age at diagnosis	**0.030**	1.034	1.003–1.065
LNI	**0.003**	2.630	1.386–4.990

LNI: lymph node involvement. Statistically significant data is highlighted in bold.

## Data Availability

The data presented in this study are available within the article and Supplementary Materials.

## Supplementary Materials

The following supporting information can be downloaded at: https://www.mdpi.com/article/10.3390/medicina62061135/s1. Table S1. Clinicopathological characteristics according to p53 status in the carcinomatous component of uterine carcinosarcoma; Table S2. Clinicopathological characteristics according to p53 status in the sarcomatous component of uterine carcinosarcoma; Table S3. Clinicopathological characteristics according to overall p53 status in uterine carcinosarcoma; Table S4. Clinicopathological characteristics according to MMR status in the carcinomatous component of uterine carcinosarcoma; Table S5. Clinicopathological characteristics according to MMR status in the sarcomatous component of uterine carcinosarcoma; Table S6. Clinicopathological characteristics according to overall MMR status in uterine carcinosarcoma.
