# Supplementary material for: Prognostic Implications of Combined p53 and Mismatch-Repair Immunophenotypes in Uterine Carcinosarcoma
Source: Medicina (Kaunas). 2026 Jun 10;62(6):1135. doi: 10.3390/medicina62061135 (PMC13302820; doi:10.3390/medicina62061135)
Supplement: Supplementary file 1 [file medicina-62-01135-s001.zip › medicina-4360421-supplementary.pdf]

Supplementary Table S1: Clinicopathological characteristics according to p53 status in the carcinomatous component of uterine carcinosarcoma.

	p53abn	p53wt	P
Age at Diagnosis, Mean. ± SD	66.0 ± 7.6	61.5 ± 11.2	0.092
BMI, Mean. ± SD	30.0 ± 7.8	28.6 ± 8.0	0.698
Parity, n (%)			0.210
None	1 (6.2%)	7 (26.9%)	
Yes	15 (93.8%)	19 (73.1%)	
Menopause Status, n (%)			1.000
Pre/Perimenopausal (M-)	1 (4.8%)	2 (6.7%)	
Postmenopausal (M+)	20 (95.2%)	28 (93.3%)	
Comorbidity, n (%)			0.957
None	6 (28.6%)	10 (33.3%)	
Yes	15 (71.4%)	20 (66.7%)	
LVI, n (%)			0.454
Negative	7 (33.3%)	6 (20.0%)	
Positive	14 (66.7%)	24 (80.0%)	
Lymph Node Involvement, n (%)			0.957
Negative	15 (71.4%)	20 (66.7%)	
Positive	6 (28.6%)	10 (33.3%)	
Stage, n (%)			0.484
1A	6 (28.6%)	3 (10.0%)	
1B	6 (28.6%)	11 (36.7%)	
2	1 (4.8%)	3 (10.0%)	
3	6 (28.6%)	11 (36.7%)	
4	2 (9.5%)	2 (6.7%)	
Epithelial Component, n (%)			**0.025**
Endometrioid grade 3	8 (38.1%)	20 (66.7)	
Serous carcinoma	10 (47.6%)	4 (13.3)	
Others	3 (14.3%)	6 (20.0)	
Myometrial Invasion, n (%)			**0.040**
<50% or Polyp	9 (42.9%)	4 (13.3)	
≥50%	12 (57.1%)	26 (86.7)	
Status, n (%)			0.651
Alive	9 (42.9%)	16 (53.3%)	
Exitus	12 (57.1%)	14 (46.7%)	

BMI: body mass index; LVI: lymphovascular invasion; LNI: lymph node involvement; SD: standard deviation

Supplementary Table S2: Clinicopathological characteristics according to p53 status in the sarcomatous component of uterine carcinosarcoma.

	p53abn	p53wt	P
Age at Diagnosis, Mean. ± SD	64.2 ± 7.6	62.7 ± 12.0	0.594
BMI, Mean. ± SD	30.0 ± 7.5	28.5 ± 8.4	0.528
Parity, n (%)			1.000
None	4 (21.1%)	4 (17.4%)	
Yes	15 (78.9%)	19 (82.6%)	
Menopause Status, n (%)			1.000
Pre/Perimenopausal (M-)	1 (4.0%)	2 (7.7%)	
Postmenopausal (M+)	24 (96.0%)	24 (92.3%)	
Comorbidity, n (%)			0.836
None	7 (28.0%)	9 (34.6%)	
Yes	18 (72.0%)	17 (65.4%)	
LVI, n (%)			0.172
Negative	9 (36.0%)	4 (15.4%)	
Positive	16 (64.0%)	22 (84.6%)	
Lymph Node Involvement, n (%)			0.836
Negative	18 (72.0%)	17 (65.4%)	
Positive	7 (28.0%)	9 (34.6%)	
Stage, n (%)			0.643
1A	6 (24.0%)	3 (11.5%)	
1B	9 (36.0%)	8 (30.8%)	
2	2 (8.0%)	2 (7.7%)	
3	6 (24.0%)	11 (42.3%)	
4	2 (8.0%)	2 (7.7%)	
Epithelial Component, n (%)			0.065
Endometrioid grade 3	13 (52.0%)	15 (57.7%)	
Serous carcinoma	10 (40.0%)	4 (15.4%)	
Others	2 (8.0%)	7 (26.9%)	
Myometrial Invasion, n (%)			0.172
<50% or Polyp	9 (36.0%)	4 (15.4%)	
≥50%	16 (64.0%)	22 (84.6%)	
Status, n (%)			0.672
Alive	11 (44.0%)	14 (53.8%)	
Exitus	14 (56.0%)	12 (46.2%)	

BMI: body mass index; LVI: lymphovascular invasion; LNI: lymph node involvement; SD: standard deviation

Supplementary Table S3: Clinicopathological characteristics according to overall p53 status in uterine carcinosarcoma.

	p53abn	p53wt	P
Age at Diagnosis, Mean. ± SD	64.6 ± 7.5	62.0 ± 12.3	0.384
BMI, Mean. ± SD	30.2 ± 7.5	28.0 ± 8.4	0.339
Parity, n (%)			1.000
None	4 (19.0%)	4 (19.0%)	
Yes	17 (81.0%)	17 (81.0%)	
Menopause Status, n (%)			0.916
Pre/Perimenopausal (M-)	1 (3.7%)	2 (8.3%)	
Postmenopausal (M+)	26 (96.3%)	22 (91.7%)	
Comorbidity, n (%)			0.557
None	7 (25.9%)	9 (37.5%)	
Yes	20 (74.1%)	15 (62.5%)	
LVI, n (%)			0.298
Negative	9 (33.3%)	4 (16.7%)	
Positive	18 (66.7%)	20 (83.3%)	
Lymph Node Involvement, n (%)			1.000
Negative	19 (70.4%)	16 (66.7%)	
Positive	8 (29.6%)	8 (33.3%)	
Stage, n (%)			0.918
1A	6 (22.2%)	3 (12.5%)	
1B	9 (33.3%)	8 (33.3%)	
2	2 (7.4%)	2 (8.3%)	
3	8 (29.6%)	9 (37.5%)	
4	2 (7.4%)	2 (8.3%)	
Epithelial Component, n (%)			0.062
Endometrioid grade 3	13 (48.1%)	15 (62.5%)	
Serous carcinoma	11 (40.7%)	3 (12.5%)	
Others	3 (11.1%)	6 (25.0%)	
Myometrial Invasion, n (%)			0.298
<50% or Polyp	9 (33.3%)	4 (16.7%)	
≥50%	18 (66.7%)	20 (83.3%)	
Status, n (%)			0.330
Alive	11 (40.7%)	14 (58.3%)	
Exitus	16 (59.3%)	10 (41.7%)	

BMI: body mass index; LVI: lymphovascular invasion; LNI: lymph node involvement; SD: standard deviation

Supplementary Table S4: Clinicopathological characteristics according to MMR status in the carcinomatous component of uterine carcinosarcoma

	MMR-intact	MMR-deficient	P
Age at Diagnosis, Mean. ± SD	63.7 ± 10.1	62.3 ± 10.0	0.656
BMI, Mean. ± SD	29.1 ± 7.7	29.4 ± 8.8	0.917
Parity, n (%)			0.718
None	5 (16.1%)	3 (27.3%)	
Yes	26 (83.9%)	8 (72.7%)	
Menopause Status, n (%)			0.773
Pre/Perimenopausal (M-)	3 (7.7%)	0 (0.0%)	
Postmenopausal (M+)	36 (92.3%)	12 (100.0%)	
Comorbidity, n (%)			0.999
None	12 (30.8%)	4 (33.3%)	
Yes	27 (69.2%)	8 (66.7%)	
LVI, n (%)			0.238
Negative	12 (30.8%)	1 (8.3%)	
Positive	27 (69.2%)	11 (91.7%)	
Lymph Node Involvement, n (%)			0.217
Negative	29 (74.4%)	6 (50.0%)	
Positive	10 (25.6%)	6 (50.0%)	
Stage, n (%)			0.337
1A	8 (20.5%)	1 (8.3%)	
1B	14 (35.9%)	3 (25.0%)	
2	4 (10.3%)	0 (0.0%)	
3	10 (25.6%)	7 (58.3%)	
4	3 (7.7%)	1 (8.3%)	
Epithelial Component, n (%)			0.261
Endometrioid grade 3	20 (51.3%)	8 (66.7%)	
Serous carcinoma	13 (33.3%)	1 (8.3%)	
Others	6 (15.4%)	3 (25.0%)	
Myometrial Invasion, n (%)			0.238
<50% or Polyp	12 (30.8%)	1 (8.3%)	
≥50%	27 (69.2%)	11 (91.7%)	
Status, n (%)			0.285
Alive	17 (43.6%)	8 (66.7%)	
Exitus	22 (56.4%)	4 (33.3%)	

BMI: body mass index; LVI: lymphovascular invasion; LNI: lymph node involvement; SD: standard deviation.

Supplementary Table S5: Clinicopathological characteristics according to MMR status in the sarcomatous component of uterine carcinosarcoma.

	MMR-intact	MMR-deficient	P
Age at Diagnosis, Mean. ± SD	64.0 ± 9.9	61.0 ± 10.6	0.406
BMI, Mean. ± SD	29.1 ± 7.5	29.6 ± 9.7	0.862
Parity, n (%)			0.452
None	5 (15.2%)	3 (33.3%)	
Yes	28 (84.8%)	6 (66.7%)	
Menopause Status, n (%)			0.895
Pre/Perimenopausal (M-)	3 (7.3%)	0 (0.0%)	
Postmenopausal (M+)	38 (92.7%)	10 (100.0%)	
Comorbidity, n (%)			0.999
None	13 (31.7%)	3 (30.0%)	
Yes	28 (68.3%)	7 (70.0%)	
LVI, n (%)			0.396
Negative	12 (29.3%)	1 (10.0%)	
Positive	29 (71.7%)	9 (90.0%)	
Lymph Node Involvement, n (%)			0.783
Negative	29 (71.7%)	6 (60.0%)	
Positive	12 (29.3%)	4 (40.0%)	
Stage, n (%)			0.768
1A	8 (19.5%)	1 (10.0%)	
1B	14 (34.1%)	3 (30.0%)	
2	4 (9.8%)	0 (0.0%)	
3	12 (29.3%)	5 (50.0%)	
4	3 (7.3%)	1 (10.0%)	
Epithelial Component, n (%)			0.061
Endometrioid grade 3	21 (51.2%)	7 (70.0%)	
Serous carcinoma	14 (34.1%)	0 (0.0%)	
Others	6 (14.6%)	3 (30.0%)	
Myometrial Invasion, n (%)			0.396
<50% or Polyp	12 (29.3%)	1 (10.0%)	
≥50%	29 (70.7%)	9 (90.0%)	
Status, n (%)			0.067
Alive	17 (41.5%)	8 (80.0%)	
Exitus	24 (58.5%)	2 (20.0%)	

BMI: body mass index; LVI: lymphovascular invasion; LNI: lymph node involvement; SD: standard deviation.

Supplementary Table S6: Clinicopathological characteristics according to overall MMR status in uterine carcinosarcoma.

	MMR-intact	MMR-deficient	P
Age at Diagnosis, Mean. ± SD	63.7 ± 10.1	62.2 ± 10.0	0.658
BMI, Mean. ± SD	29.1 ± 7.7	29.4 ± 8.8	0.693
Parity, n (%)			0.718
None	5 (16.1%)	3 (27.3%)	
Yes	26 (83.9%)	8 (72.7%)	
Menopause Status, n (%)			0.773
Pre/Perimenopausal (M-)	3 (7.7%)	0 (0.0%)	
Postmenopausal (M+)	36 (92.3%)	12 (100.0%)	
Comorbidity, n (%)			1.000
None	12 (30.8%)	4 (33.3%)	
Yes	27 (69.2%)	8 (66.7%)	
LVI, n (%)			0.238
Negative	12 (30.8%)	1 (8.3%)	
Positive	27 (69.2%)	11 (91.7%)	
Lymph Node Involvement, n (%)			0.217
Negative	29 (74.4%)	6 (50.0%)	
Positive	10 (25.6%)	6 (50.0%)	
Stage, n (%)			0.260
1A	8 (20.5%)	1 (8.3%)	
1B	14 (35.9%)	3 (25.0%)	
2	4 (10.3%)	0 (0.0%)	
3	10 (25.6%)	7 (58.3%)	
4	3 (7.7%)	1 (8.3%)	
Epithelial Component, n (%)			0.227
Endometrioid grade 3	20 (51.3%)	8 (66.7%)	
Serous carcinoma	13 (33.3%)	1 (8.3%)	
Others	6 (15.4%)	3 (25.0%)	
Myometrial Invasion, n (%)			0.238
<50% or Polyp	12 (30.8%)	1 (8.3%)	
≥50%	27 (69.2%)	11 (91.7%)	
Status, n (%)			0.285
Alive	17 (43.6%)	8 (66.7%)	
Exitus	22 (56.4%)	4 (33.3%)	

BMI: body mass index; LVI: lymphovascular invasion; LNI: lymph node involvement; SD: standard deviation.
